# Is It Important to Perform Physical Activity During Coronavirus Pandemic (COVID-19)? Driving Action for a Correct Exercise Plan

**DOI:** 10.3389/fpubh.2020.602020

**Published:** 2020-11-02

**Authors:** Valentina Natalucci, Vittoria Carnevale Pellino, Elena Barbieri, Matteo Vandoni

**Affiliations:** ^1^Division of Exercise and Health Sciences, Department of Biomolecular Science, University of Urbino Carlo Bo, Urbino, Italy; ^2^Laboratory of Adapted Motor Activity (LAMA), Department of Public Health, Experimental and Forensic Medicine, University of Pavia, Pavia, Italy; ^3^Department of Industrial Engineering, University of Tor Vergata, Rome, Italy

**Keywords:** exercise, physical activity guidelines, health education, COVID-19, global emergency

Coronavirus disease (COVID-19) continues to outbreak all over the world causing a public health concern (1) and on March 11th, the pandemic was declared a public health emergency of international proportions by World Health Organization (WHO). To limit viral transmission, most governments introduced national quarantine reducing movements and social interaction with the imperative “stay at home.” Lockdown is considered the extreme option to protect health and in particularly most vulnerable people (those aged ≥65 years, and those with pathologies such as chronic heart, lung kidney and liver diseases, diabetes, and obesity), but it caused considerable disruption to the routine of the general population (2, 3). The closure of gyms, stadiums, pools, dance and fitness studios, physiotherapy centers, parks, playgrounds reduced the possibility to practice Physical Activity (PA) in outdoor setting and, in general, the exercise options (4, 5). Although PA and exercise management in autonomy is challenging, some authors suggest that exercise and regular PA are useful to help ease anxiety and to improve mental health and general well-being (6, 7). Moreover, Nieman and Wentz (8) showed the beneficial role of PA in disease prevention, as an adjuvant treatment in chronic diseases with a protective effect on the immune system, whose optimal status is crucial to respond adequately to the threat of COVID-19 (8). In this Opinion article, we analyze possible adaptations of the exercise guidelines to the current pandemic emergency, proposing an Exercise Schedule to prevent or mitigate the physical inactivity (9–11).

## Guidelines for Physical Activity for Health Promotion

During the initial phase of COVID-19 Pandemic (12), to ensure an optimal health status and maintain well-being and fitness, guidelines established the minimal amount of PA to practice. The WHO established guidelines on the minimal amount of PA to maintain well-being and fitness. For adults, it is recommended at least 150 min of moderate-intensity PA or 75 min of vigorous-intensity PA, including muscle-strengthening activities twice weekly (5, 8–11).

## COVID-19 Emergency: the Ways to be Physically Active

PA recommendations were implemented to promote an active lifestyle and improve global health. In particular, Chen et al. (13) recommended regular PA to maintain healthy physical and immune system functions in an unsafe environment and suggested various reliable, simple, and easily implementable exercises. As well as, Ricci et al. (14) proposed specific recommendations and tips for Home-Based PA management. However, COVID-19 pandemic highlighted the importance of understanding common barriers to PA practice and contrast sedentary creating strategies both for exercise specialists and general population. PA in healthy individuals can be self-directed or supervised by exercise professionals (such as trainers, specialists in sport sciences, kinesiologists) that are conscious of exercise prescription FITT-VP principle [i.e., frequency (F), intensity (I), time (T), type (T), volume (V), and progression (P) over time]. In fact, it is crucial to differentiate the concept of “exercise” compared to “PA”: exercise is a subset of planned, structured and repeated PA with the aim of improve or maintain physical fitness. People, during the COVID-19 lockdown, tried to contrast sedentary behavior with various strategies without the use of specialized technology and equipment (i.e., brisk walking, stair climbing, yard or house-work and playing active). Nevertheless, the use of exercise technologies evolved to facilitate and support people in PA practice involving people, coaches and clinicians with adequate support and education. In fact, the virtual environment is attractive and could be a safe and reliable solution to contrast sedentary. There are many platforms or applications free of charge that help people to maintain healthy habits and an active lifestyle.

## A Potential New Way to Promote an Active Lifestyle

The role of exercise specialists in the community and health care system is increasingly important, as well as a way to promote a strategy for driving healthy community to safely and effectively prevent most chronic illness through an active lifestyle. For this reason, we have developed an Exercise Schedule focused on practical applications and recommendations to help people to provide people with guidelines to help them exercise by themselves. Exercise specialists, following FITT-VP supported principles will be able to adapt the Exercise Schedule to peculiar capacities of people and different training environments. The home-based exercise programs can be delivered by exercise specialists exploiting the using telehealth (or telemedicine), i.e., the remote delivery of health care as well as a range of other services such as wellness promotion through technology (15). Moreover, the home-based exercise programs are reasonable, usually well-accepted thanks to the possibility to involve entire families during leisure time. The Exercise Schedule presented in Table 1. is addressed to healthy people without any diagnosis of illness and, considering the actual exercise prescription guidelines (16), suggests a mixed (aerobic and resistance) training program. Preliminary to engage any fitness program, sedentary people and those with health issues or concerns may need to consult with medical staff before starting a new exercise routine. However, the American College of Sports Medicine recommendations simplify the pre-screening process by eliminating the need for medical clearance and/or exercise testing in many individuals, especially when low-to moderate intensity exercise is contemplated.

**Table 1:** Exercise schedule.

**PA** **self-report**	**Perceived exertion rating** **(6–20 RPE scale)**		**Intensity**	**Absolute intensity**	**Example of home-and-outdoor exercise (Healthy adults** **≥18 years)**				
**IPAQ zone**	**RPE**	**Level of exertion**	**Level zone**	**METs**	**Cardiorespiratory endurance exercise**	**Resistance (Strength) Exercise**			
						**Body zone**	**Exercise**	**Exercise per level of intensity**	**Assisted equipment or resistance applied**
Inactive (I) (<600 MET-Min week-1)	<9	Very light	Very light	<3.0	Walking, 1.7 mph (2.7 km/h), level ground (2.3 METs); Walking, 2.5 mph (4 km/h) (2.9 METs) Bicycling, stationary, 50 watts (3.0 METs); Calisthenics, home exercise, light or moderate effort (3.5 METs)	Upper body	Push-ups	LI: Wall push-ups	Mat
								LMA: Push-ups on knees	
								LA: Basic Push-ups (or advanced)	
	9–11	Very light-fairly light	Light	3.1–4.0			Triceps extension	LI: Low	Elastic band (Therabands)
								LMA: Medium	
								LA: High	
							Shoulder press	LI: Low	Elastic band (Therabands)
								LMA: Medium	
								LA: High	
Moderate Active (MA) (≥600 as <3000 MET-Min week-1)	12–14	Fairly light to somewhat hard	Moderate	3.0–5.9	Bicycling, stationary, 100 watts (5.5 METs); Walking 3.0 mph (4.8 km/h) (3.3 METs); Calisthenics, home exercise, light or moderate effort (3.5 METs); Walking 3.4 mph (5.5km/h) (3.6 METs)	Lower body	Squats	LI: Bodyweight squat	Mat
								LMA: Squat jump	
								LA: Single leg squat	
							Walking lunges	LI: Slider revers lunge	Mat
								LMA: Pendulum lunge	
								LA: Skater squat	
							Side-lying hip abduction	LI: Low	Elastic band (Therabands)
								LMA: Medium	
								LA: High	
							Calf-raise	LI: Seated calf raise	Mat
								LMA: Single-leg standing calf raise with assistance	
								LA: Sigle-leg standing calf raise	
							Bridge	Li: Basic bridge (two legs)	Elastic band (Therabands)
								LMA: Single leg bridge	
								LA: Swiss ball bridge	
Active (A) (≥3000 MET-Min week-1)	15–17	Somewhat hard to very hard	Vigorous	6.0–8.7	Jogging, general (7.0 METs); Calisthenics (e.g., push-ups, sit-ups, pullups, jumping jacks), heavy, vigorous effort (8.0 METs); Running jogging, in place (8.0 METs)	Abdominal and core	Crunches	LI: Basic crunches LMA: Crunches reverse	Mat
								LA: Bicycle crunches	
							Plank	LI: Plank with both Knees Bent	Mat
								LMA: Plank full	
								LA: Plank with legs lifts (or advanced)	
							Bird dog	LI: Twisted bird dog	Mat
								LMA: Basic bird dog	
								LA: Bird dog pushup	

MET-min, metabolic equivalents (MET) of energy expenditure for a physical activity performed for a given number of minutes (min), calculated as MET × mins; LI, Level Inactive; LMA, Level Moderate Active; LA, Level Active.

To better adapt our program to individual training level, we suggest to self-evaluate PA level through the International Physical Activity Questionnaire (IPAQ) (17) prior to approaching the training and to manage the intensity with Rate Perceived Exertion (RPE) through the Borg RPE Scale (18). The Borg Scale takes into account fitness level: it runs from 6 to 20 and multiplying the Borg score by 10 gives an approximate heart rate for a particular level of activity. Aerobic training should be performed 3–5 times per week while resistance training 2–3 times per week. In general, people could improve duration, intensity and difficulty of training when they feel confident with their routine. Our program provides aerobic activities controlled for intensity by RPE for inactive (6–11 RPE), moderate active (12–14 RPE) and active people (15–17). We excluded to train people with higher levels of RPE for the risk to reach anaerobic intensities. Moreover, exercises were selected following Metabolic Equivalent Tasks (METs) intensities (19) and take into account initial evaluation of self-reported PA level. The duration of each training session should last from 20 (Inactive people) till to 60 (Active people) min. Resistance training was designed specifying body zones and with different modalities of execution. To help people and facilitate exercise execution, we suggested elastic resistance training (ERT) for some exercises. Its benefits include improved functional capacity, increased strength and endurance and represent safe equipment during home-confinement (20). Exercises should be repeated from 8 to 12 times per session. Finally, a warm-up and a cool-down phase for every training session should include a 5–10 min routine of neuromuscular, mobility and flexibility for the main muscular groups (21).

## Translation to Health Education Practice

This document, through an Exercise Schedule, provides information and practical applications to drive people to achieve PA guidelines recommendations. Many people need to increase their activity level and adopt a more active lifestyle. Technology can play a role in providing them the information and support the need to do so. Improving people's health will ameliorate some health issues and reduce health care costs. This is particularly needed given the impact and costs of the current COVID-19 Pandemic on the health care system. The challenge for exercise specialists is to provide safe and effective exercise programs that people will adhere to in the long term. We are strongly convinced that using the present information to manage types, duration, and intensities of exercise will permit people to enrich PA experience and to achieve better health outcomes.

## Author Contributions

VN: conceptualization, wrote—original draft, review and editing, and planned and project training protocol. VC: wrote—original draft and planned and project training protocol. EB: supervision. MV: conceptualization, wrote—original draft, review and editing, and supervision. All authors contributed to the article and approved the submitted version.

## Conflict of Interest

The authors declare that the research was conducted in the absence of any commercial or financial relationships that could be construed as a potential conflict of interest.
